# Conjugated Polymers Containing Building Blocks 1,3,4,6-Tetraarylpyrrolo[3,2-b]pyrrole-2,5-dione (isoDPP), Benzodipyrrolidone (BDP) or Naphthodipyrrolidone (NDP): A Review

**DOI:** 10.3390/polym11101683

**Published:** 2019-10-15

**Authors:** Zhifeng Deng, Taotao Ai, Rui Li, Wei Yuan, Kaili Zhang, Huiling Du, Haichang Zhang

**Affiliations:** 1 National and Local Joint Engineering Laboratory for Slag Comprehensive Utilization and Environmental Technology, School of Material Science and Engineering, Shaanxi University of Technology, Hanzhong 723001, China; dengzf@snut.edu.cn (Z.D.); aitaotao0116@126.com (T.A.); zkl1587040740@163.com (K.Z.); 2 Key Laboratory of Rubber–Plastic of Ministry of Education (QUST), School of Polymer Science and Engineering, Qingdao University of Science and Technology, 53 Zhengzhou Road, Qingdao 266042, China; Lirui950123@163.com; 3 School of Materials Science and Engineering, Xi’an University of Science and Technology, Xi’an 710054, China; yuanwei755918@163.com

**Keywords:** conjugated polymers, isoDPP, benzodipyrrolidone, naphthodipyrrolidone

## Abstract

π-Conjugated organic donor–acceptor (D–A) type polymers are widely developed and used in electronic device. Among which, diketopyrrolopyrrole (DPP)-based polymers have received the most attention due to their high performances. The novel chromophores named 1,3,4,6-tetraarylpyrrolo[3,2-b]pyrrole-2,5-dione (isoDPP), benzodipyrrolidone (BDP) and naphthodipyrrolidone (NDP) are resemble DPP in chemical structure. IsoDPP is an isomer of DPP, with the switching position of carbonyl and amide units. The cores of BDP and NDP are tri- and tetracyclic, whereas isoDPP is bicyclic. π-Conjugation extension could result polymers with distinct optical, electrochemical and device performance. It is expected that the polymers containing these high-performance electron-deficient pigments are potential in the electronic device applications, and have the potential to be better than the DPP-based ones. IsoDPP, BDP, and NDP based polymers are synthesized since 2011, and have not receive desirable attention. In this work, the synthesis, properties (optical and electrochemical characteristics), electronic device as well as their relationship depending on core-extension or structure subtle optimization have been reviewed. The final goal is to outline a theoretical scaffold for the design the D–A type conjugated polymers, which is potential for high-performance electronic devices.

## 1. Introduction

**Figure 1:**
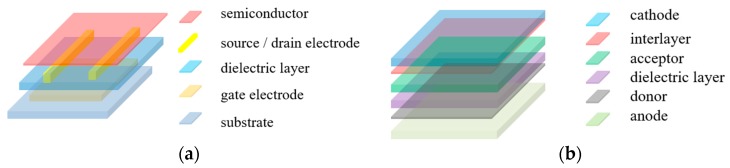
Schematic representation of an organic filed-effect transistors (**a**) and organic solar cells (**b**).

Organic π-conjugated polymers are promising candidates for electronics device applications due to light weight, low cost and potential use in flexible devices, such as organic filed-effect transistors (OFETs), organic photovoltaic (OPV) solar cells, etc. [1,2,3]. The OFET is fundamental building block of modern electronic devices and used to amplify and switch electronic signals. The OTFT operation depends on the charge (electron or hole) carrier accumulation in the semiconductor enabled by applying a bias on gate. If the gate voltage is smaller than the threshold voltage (*V*_t_), there is no free charge carriers in the conducting channel and the source−drain current will be near zero. However, once the applied gate voltage is over *V*_t_, charges will be created in close vicinity of the semiconductor/dielectric interface, and as a consequence, the source-drain current (*I*_ds_) will dramatically increase when a voltage (*V*_sd_) is applied between them [4]. The architectures of OFETs are shown in Figure 1a. As a cost-free and environmental friendly energy source, organic solar cells (OSCs) have shown significantly potential for generating electrical energy directly from sunlight using photo-voltaic (PV) technologies. Universally, the OSCs absorb the photons from the sun, meanwhile, intermolecular excisions are generated. Subsequently, the excitons diffuse to the donor/acceptor semiconductor interfaces and dissociate via an electron-transfer process to form a geminate pair. Eventually, the separated charge carriers (electron and hole) migrate to their respective electrodes under internal electric field and are collected at the electrodes [4]. The architectures of OPV are shown in Figure 1b.

**Figure 2:**
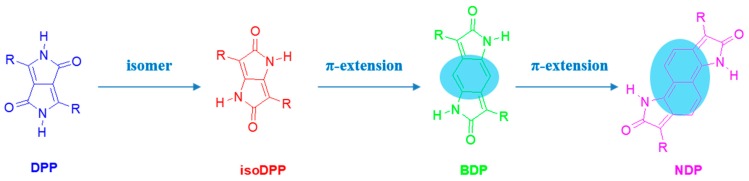
Chemical structure of diketopyrrolopyrrole (DPP), 1,3,4,6-tetraarylpyrrolo[3,2-b]pyrrole-2,5-dione (isoDPP), benzodipyrrolidone (BDP), and naphthodipyrrolidone (NDP).

In the past few years, a growing number of chemists focus their attention on development new π-conjugated polymers for electronics applications [5,6,7,8,9,10,11,12]. Some of the most widely studied polymers are donor–acceptor (D–A) type polymers due to its intrinsic properties such as high charge transfer mobility. Among a wide variety of acceptors, diketopyrrolopyrrole (DPP) receives lots of attention and have been widely used as a comonomer in large amount for polymer synthesis [13,14,15,16,17]. The designed polymers often show excellent performance. Altering the position of carbonyl and amide units of the DPP pigment result an isomer of DPP, named 1,3,4,6-tetraarylpyrrolo[3,2-b]pyrrole-2,5-dione (isoDPP, Figure 2). IsoDPP chromophore exhibit deep color, weather resistance, strong π-stacking, high electron mobility (μ_e_), and good chemical-, thermal-, and photo-stability [18].

Chromophore with π-conjugation extension could not only enlarge the charge transfer pathway, but also improve its optical and electrochemical properties. It is often important to use large π-conjugation units to build polymers for good electronic device performance. The molecular structures of BDP and NDP resemble isoDPP except that the BDP and NDP core are tri- and tetracyclic, whereas isoDPP is bicyclic (Figure 2). The polymers based on isoDPP, BDP, and NDP show broad UV/vis absorption in the visible and low band gap, which reported since 2011 [19,20,21]. To the best of the knowledge of the authors, only a few articles have reported these chromophores. Using these high-performance pigments to construct polymers, which should show as good performance as DPP ones, even improvement regarding to the photostability and charge transfer mobility. This is mainly attributed to the following factors: (i) The presence of heteroatoms (nitrogen and oxygen atom) in the central rings among the compounds favors intermolecular interaction, which will enhance the charge transport; (ii) the compounds contain bisamide (or bislactam) acceptor with a quinoid structure in its electronic ground state., lowering semiconductor lowest unoccupied molecular orbitals (LUMOs), and thus facilitating and stabling electrons injection [22]; (iii) the solubility of the compounds can be adjusted through *N*-amide substituents, which can offer excellent solution processability, well polymerization and fine-tuned film morphology; (iv) the cohromophores are deep color, which absorb light in the visible region with high extinction coefficients; and (v) the substitution of an aromatic core with two sets of π-accepting imides group led the chromophores electron deficient. Though these chromophores have numerous advantages, they have not received desirable attention. In this review, the synthesis, properties (optical and electrochemical characteristics), electronic device as well as their relationship depending on core-extension or structure subtle optimization have been reviewed. In addition, a future-outlook is also pointed out. 

## 2. Small Molecules and Monomers

### 2.1. IsoDPP

**Scheme 1:**
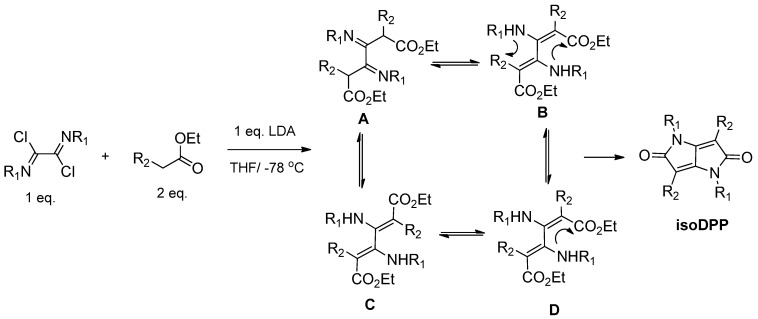
Synthesis of isoDPP derivate. (Reprinted with permission from Ref. [18]).

As a natural dye, isoDPP is a regioisomer of DPP with switching position of the carbonyl group and the nitrogen atom (Figure 2). Compared with DPP, the ketone and *N*-alkyl position of isoDPP are interchanged causing the electron withdrawing functional groups closed to the conjugation pathway and lowering the highest occupied molecular orbital (HOMO) level, improving the stability of the electronic device. IsoDPP was firstly synthesized roughly 30 years ago in one step from pluvinic acid using relatively harsh reaction conditions (autoclave reaction, 140–180 °C) [23], or three steps starting from (*N*-phenylacetyl)acetic acid amino ester [24]. However, the important physical properties of the isoDPPs, such as brilliant red color and high melting points were unnoticed. In 1997, J. Wuckelt et al. took notice of this compound and found an efficient strategy to synthesize isoDPP in a single reaction step with a high yield of up to 75% when a benzene unit substituted R1 and R2 (Scheme 1) [18]. The formation of product isoDPP involves two-fold attack of 2 equivalent of the ester enolate onto the bis(imidoyl)chloride to obtain the open-chained intermediate A, which rapidly equilibrates with the enamine-tatuometres B, C, and D. P. Langer further proved this method [25]. 

**Figure 3:**
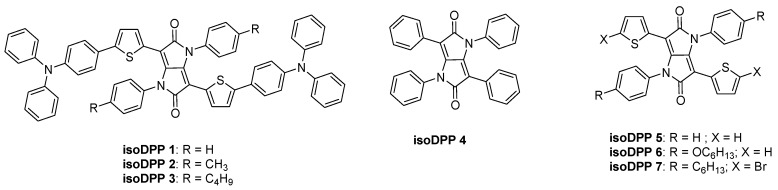
Structures of small molecular based on isoDPP.

Subsequent studies concentrated on the preparation of derivatives with different substituents. Some research put their attention on isoDPP flanked with thienyls. IsoDPP based compound exhibited extremely weak fluorescence down to 2 × 10^−4^ in solution [26], while the DPP molecules showed very high fluorescence up to 0.88 [27]. M. Kirkus et al. explained the low quantum yields compared with DPP ones, which can be ascribed to a different symmetry of the lowest singlet excited state (S_1_) in isoDPP and the corresponding loss in oscillator strength of the lowest energy transition. [26] This implies that the S_1_ to ground state (S_0_) of isoDPP is a forbidden transition and efficient non-radiative decay pathways of the excited state. In this study, it was determined that the isoDPP chromohphore exhibited not only broad absorption peak in the visible, but also with a weak absorption tail at longer wavelength length. The highly absorption in the visible and weak emission properties of isoDPP endow this chromophore potential in photovoltaic device. H. Suh and coworkers reported three small molecules containing isoDPP as acceptor and ended with thienyl-triphenylamine unit as donor (isoDPP1-3, Figure 3) [28]. The present study indicated that the three molecules exhibit broad absorption peak around 510 nm and low band gap (1.66 eV). Device comprising isoDPP 3 and PC71BM (1:4) exhibited a good open-circuit voltage (V_oc_) of 0.79 V, a short circuit current (J_sc_) of 6.0 mA/cm^2^, and power conversion efficiency (PCE) of 1.56%. Up to now, this article is the single one to study the OPV device based on isoDPP small molecule. 

In order to study the isoDPP molecular packing properties, D. Gendron et al. [29] and H. Zhang et al. [30] prepared the single crystal of small isoDPP molecules flanked with phenyl or thienyl unit attached aromatic group (isoDPP 4 to 7, Figure 3). The core of isoDPP was fully coplanar as DPP, which ensured full conjugation. Using thiophene ring (isoDPP 5) instead of phenyl ring (isoDPP 4, Figure 3) attached the isoDPP core was a crucial effect on the decreasing the inter-plane distance (from ketone oxygen to ketone) by reducing the twisting angle. The alkoxy or alky chains-substituted phenyl ring attached to the nitrogen atom (isoDPP 6 and 7, Figure 3) does not promote π–π direct interaction, while there were strong π–π interactions for DPP-type molecules [31].

**Scheme 2:**
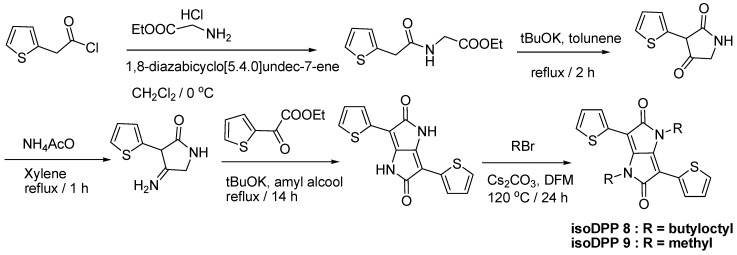
Synthesis of *N*-alkylated isoDPP derivate isoDPP 8 and 9. (Reprinted with permission from Ref. [32]).

In 2013, S. Lu et al. [32] discovered a new strategy to synthesize *N*-alkylated isoDPP (isoDPP 8 and 9) in five steps, however, it turned out the yield of this product was extremely low (10–18%, Scheme 2). The *N*-alkylated isoDPP was planar in the solid state with torsional angle between the thiohene units and isoDPP core being 8.7° determined by crystal structure analysis. This value was 16° and 1.5 ° smaller than isoDPP 6 and DPP with similar structure respectively [29,33]. The core-core intermolecular distances of *N*-alkylated isoDPP was very small being 3.34 Å, while the DPP chromophore was around 3.51 Å and *N*-phenyl isoDPP was 6.97 Å. This indicates that *N*-alkyl chain replaced *N*-phenyl unit of isoDPP core could improve the planarity and π-stacking of the chromophore, as well as lower the band gap, which was beneficial for the charge transfer within the single molecule or between neighboring molecules. 

### 2.2. Benzodipyrrolidone

**Scheme 3:**
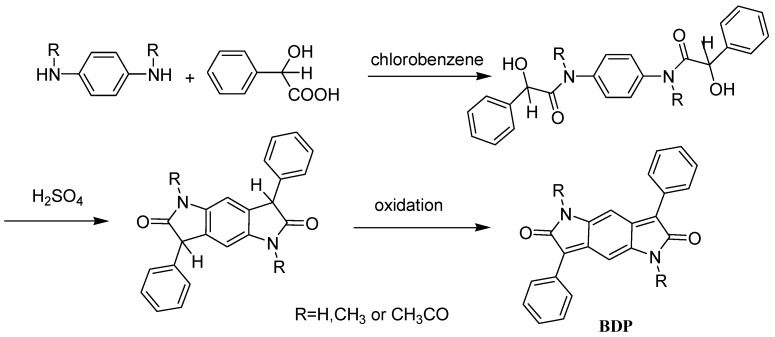
Synthesis of a phenyl-flanked BDP derivate. (Reprinted with permission from Ref. [34]).

Benzodipyrrolidone was first designed and synthesized by Greenhalgh et al. through three steps with a good yield (Scheme 3) [34,35]. This chromophore exhibits a good solubility in common organic solvents after *N*-alkylation with a high extinction coefficient, which was used as a colorant due to its deep color and good thermal stability and photostability. The quinodimethane moiety in BDP, in analogy to the tetracyanoquinodimethane (TCNQ), is expected to exhibit extensive π–π stacking. 

**Figure 4:**
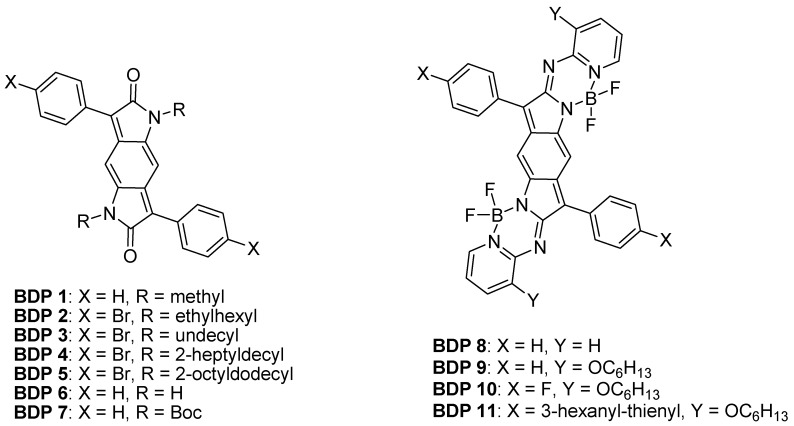
Structures of small molecular based on phenyl-flanked BDP (**left**) and BDP-based aza-BODIPY (**right**) derivate.

In 2011, W. Cui et al. noticed this pigment and firstly used it as comonomer for polymerization [25]. In this article, the solid-state structure of phenyl-flanked BDP (BDP 1, Figure 4) was analyzed through X-ray, which showed that the core of BDP was fully coplanar as DPP and isoDPP [29,33]. The dihedral angle between BDP core and the phenyl ring was 38°. BDP 2 exhibited a broad UV/vis absorption with a peak at 458 nm in solution and 523 nm in thin film. Comparing the maximum absorption in solution, the large bathochromic shift for the solid state indicated a strong intermolecular interaction. The LUMO of BDP 2 was estimated to be −3.53 eV, which was 0.35 eV lower than the corresponding DPP chromophore [36]. This indicated that BDP was a strong electron affinity pigment and could be easy reduced. To reduce the LUMO energy levels, P. Deng et al. reported a novel and effective strategy via *N*-acylation instead of *N*-alkylation, which showed the LUMO energy levels can reduce almost 0.3 eV (BDP 3 and 4, Figure 4) [37]. This method might also suitable for other pigments containing lactam unit.

For pigments, the stability is an important characteristic for its application. H. Zhang et al. [38] reported that BDP 5 (Figure 4) exhibited very stable properties in the ultraviolet light. The initial slope the rate constant (*k*) was 4.43 × 10^−3^ (ln (*A_t_*/*A_0_*, *A_t_*_,0_ = absorbance at time *t* and time *t* = 0) vs. time min). Good, reversible and stable electrochemical properties were reported by Y. Ling et al. [39]. Upon incorporation various donors, BDP-based small molecules showed various color and cover the whole visible range. These molecules showed reversible electrochemical properties and dramatic color switching under negative potentials owing to their two-step reversible reductive processes. BDP-based small molecules were used to construct reversible and stable multicolored electrochromism device. Very soon, H. Zhang et al. functionalized *tert*-butoxylcarbonyl (*t*-Boc) unit into the BDP core (BDP-6, Figure 4) to form soluble BDP-7 [40]. Upon thermal annealing, through hydrogen bond rearrangement of molecules resulting in crystal-to-crystal transition, a hydrogen bonded crystal BDP OFET was prepared. The hydrogen bonded device exhibited p-type characteristic with hole mobility (μ_h_) of 0.084 cm^2^ V^−1^ s^−1^, while the μ_h_ of BDP-7 was only 1.8 × 10^−3^ cm^2^ V^−1^ s^−1^. This study indicated that the carbonyl unit and amide group of the BDP core could form hydrogen bonds, which resulted the molecules self-assemble to form more ordered structure. Materials with ordered packing often showed good charge carrier mobility and conductivity [41,42].

**Scheme 4:**
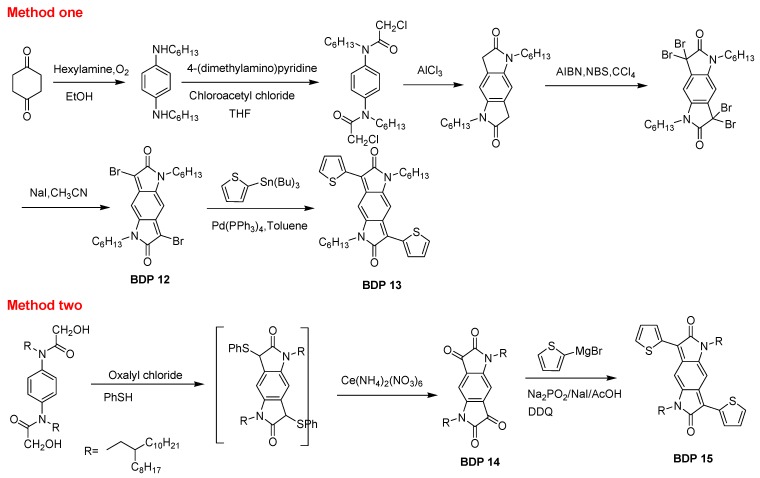
Two different methods to synthesize thienyl-flanked BDP derivate. (Reprinted with permission from Ref. [43,44]).

To achieve the goal of optical absorption extending to the longer wavelength of BDP derivatives, thiophene-flanked BDPs through different methods (Scheme 4) were designed and synthesized. C. Wei et al. synthesized the core of BDP with bromine atoms through five steps (BDP 12, Scheme 4) [43]. J. Rumer et al. obtained bis-isatin BDP derivative (BDP 14, Scheme 4) [44]. The two different monomers can be easily substituted with thiophene, furan or other donor groups to form D–A systems within the molecules. Unfortunately, no articles reported on furan-BDP or other donor units-flanked BDPs, except BDP 13 and 15. The maximum absorption wavelength of BDP 13 was 101 nm red-shifted compared with the phenyl-flanked BDP (BDP 2, Figure 4). This is because thiophene is a more electron-rich unit compared with benzene. There was a 12.7° dihedral angle between the thiophene and BDP core, which was 25.3° smaller compared with the diphenyl analogue but 4° larger than the thiophene-flanked isoDPP [29]. It seems that thiophene-, instead of benzene-, substituted compounds can lead to more planar structure. The nearest molecular distance in BDP 13 was 3.29 Å, which was slightly smaller than both isoDPP 6 and BDP 1 [21,29]. This indicates stronger π-π interaction happened in BDP 13 than similar analogue (isoDPP 6 and BDP 1) which favors a bathochromic shift.

Y. Wang et al. reported a series of BDP-based dimeric aza-BODIPY dyes (BDP 8-11, Figure 4) with push-pull structure and large conjugated systems, and were compared with BDP [45]. This is expected to induce bathochromic shift in the absorption and distinct electrochemical properties. The dihedral angle between the pendant phenyl unit and the BDP-BODIPY core for BDP 8 was around 25°, which was 13° smaller than BDP 1 [21]. Extending coplanar structure resulted in a strong π–π stacking with a distance of 3.3 Å for BDP 9, while the same distance for BDP 1 was 3.56 Å. The stronger stacking, improvement planarity, and extended π-conjugation resulted the absorption of BDP-based aza-BODIPY dyes exhibiting two peaks between 543 and 650 nm, which was red-shifted almost 100 nm compared with BDP 2 [21]. The LUMO levels of these molecules were between −4.00 and −4.13 eV, which was approximately 0.6 eV lower than BDP 2. This study indicated extending the coplanar structure could lead compounds with stronger electron-deficient properties for electronic device. 

### 2.3. Naphthodipyrrolidone

**Scheme 5:**
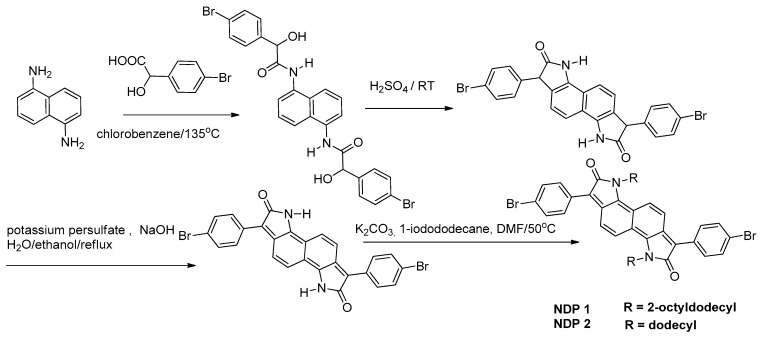
Synthesis of NDP derivate. (Reprinted with permission from Ref. [20]).

Naphthodipyrrolidone is a novel chromophore firstly developed by Zhang and Tieke [20,46]. The chemical structure of this chromophore is analogy to isoDPP and BDP (Figure 1), except the core of the NDP is tetracyclic. NDP was obtained from 1,5-naphthalenediamine with 4-bromomandelic acid in chlorobenzene followed by ring closure in sulfuric acid at room temperature. The final deep color originates from the quinonoid structure of the central core unit, which was obtained upon oxidation of the phenylene unit with potassium persulfate in good yield, typically over 70% (Scheme 5). To enhance the solubility of NDP in common organic solvents, different scale of alkylation can be used (NDP 1 and 2, Scheme 5).

H. Zhang et al. analyzed the single crystal structure of NDP 2 which showed there was a 24.9° torsional angle between the 4-bromophenyl ring and the NDP core [20]. This value was 13.1° smaller than BDP 1, indicating NDP with a more planar structure [21]. The crystal packing showed that the molecules adopt a weak, intermolecularly slipped π–π stacking separated by the distance of 3.38 Å, in other words, clearly shorter than similar analogues BDP 1 (3.56 Å) and isoDPP 4 (3.5 Å). This indicated a stronger π–π interaction was happened in NDP. The UV/vis absorption maximum of NDP was located at 567 nm, which was 219 nm and 109 nm red-shifted compared with isoDPP 4 and BDP 2 [21,29]. The bathochromic shift caused by the core extension. The NDP chromophore exhibited low fluorescence but high absorbance efficiency. In solution state, this chromophore was very stable under ambient light, but easily decomposed upon UV-light irradiation. However, BDP was stable at both conditions. Soon thereafter, Z. Deng et al. used bithiophene to substitute both side of the NDP [47]. Using obtained molecule to construct OFETs showed n-type behavior with a μ_e_ up to 0.26 cm^2^ V^−1^ s^−1^. However, similar BDP-based molecule exhibited p-type behavior with a μ_h_ of only 0.09 cm V^−1^ s^−1^. This study indicates that extension of π-conjugated system is a useful strategy to increase the performance of semiconductors. NDP is a promising chromophore for n-type semiconductor. The solid packing, UV/vis and electrochemical properties, as well as electronic device performance based on the part of the three small molecules are summarized in Table 1. 

## 3. Polymers

Materials having a delocalized electron system can absorb sunlight, create photogenerated charge carries, and transport these charge carries [48]. Compared corresponding small molecules, π-conjugated polymers normally have a bathochromic shift in the absorption and match the sunlight spectrum due to the π-extension during the polymerization. Furthermore, it also exhibits high charge mobility due to the stronger intermolecular reaction. Polymers often show higher thermal stability and photostability compared with corresponding monomers. Thus, the π-conjugated polymers, especially containing the D–A system in the structure, receive significant attention as n-type materials in bulk heterojunction (BHJ) solar cells and semiconductive materials in OFETs [49,50,51,52,53,54].

### 3.1. IsoDPP

Recently, DPP-based polymers have been widely explored and used in various electronic devices, such as OFETs, BHJ solar cells, and hole transfer materials in perovskite solar cells, among which, electronic devices have showed excellent performances [55,56,57,58,59,60]. It is expected that isoDPP polymers present similar device performance as their DPP analogues. However, compared to DPP-based polymers, isoDPP-based polymers have not been widely utilized. 

**Figure 5:**
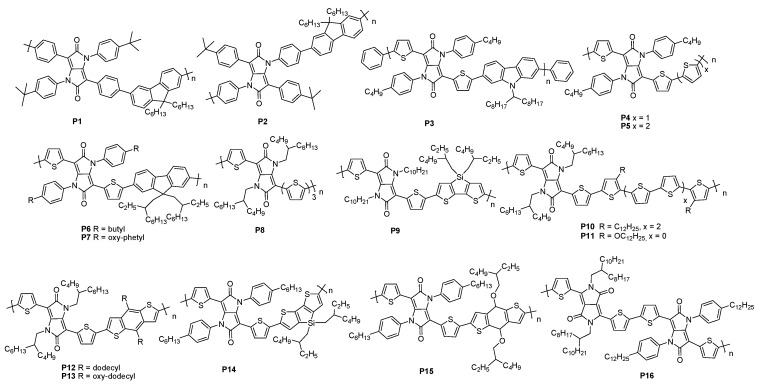
Chemical structure of polymers based on isoDPP.

In 2012, B. Tieke and co-authors reported the first isoDPP-based polymers upon Suzuki reaction between isoDPP and fluorene units (P1,P2, Figure 5) [19]. P1 and P2 are similar in chemical structure. For the polymer backbone, P1 was along the 1- and 4-position of the isoDPP core, while P2 was along 3- and 4-position. The physical UV/vis absorption properties were remarkable different between P1 and P2. The UV/vis absorption of P2 was blue-shifted by 50 nm compared with P1, which can be ascribed to the fact that π-conjugation of polymer backbone was interrupted at the lactam *N*-atoms for P2. This phenomenon also happened in the DPP-containing polymers [61]. Polymers with good π-conjugation could be obtained along 3- and 6-positions of isoDPP core. 

Carbazole is a promising electron rich unit for high-performance solar cell polymers [62,63]. S. Song et al. used carbazole as donor unit synthesized with D–A typed isoDPP-polymer (P3, Figure 5) and used it in PV device application [64]. P3 exhibited a good thermal stability with 5% weight loss of 428 °C and glass transition temperature (T_g_) occurred at 137 °C, which was similar to the copolymer containing DPP and carbazole unit in the main chain with T_g_ of 155 °C [61]. The LUMO of P3 derived from cyclic voltammetry was −3.56 eV. The UV/vis absorption maxima of P3 located at 545 and 548 nm in solution and film, respectively, which was almost 40 nm red shifted compared with similar polymers in structure containing DPP units. [65] This may be because P3 had a higher molecular weight compared with the DPP one, which favorited the bathochromic shit. The device based on P3:PCBM (1:2) blending using dichlorobenzene without thermal treatment showed a good V_oc_ of 0.82 V, a J_sc_ of 6.28 mA/cm^2^, and a fill factor (FF) of 0.39, giving a PCE of 2.0%. The performance of this device was improved compare to the one based on small molecular of isoDPP (isoDPP 4-6 exhibit PCE between 0.93% and 1.56%). R. Gironda et al. reported four polymers based on isoDPP as acceptor unit and thienyl or fluorine as donor group (P4-P7, Figure 5) [66]. P4 and P5 exhibited extremely low number molecular weight (*M_n_*) around 2 kDa while the *M_n_* of P6 and P7 were 8.2 and 13.7 kDa, respectively. The low *M_n_* could be ascribed to the poor solubility of the growing polymer caused by early precipitation during the polymerization. Atomic Force Microscopy study showed that the annealed surface of P4/PC61BM film was smooth and homogeneous. The device based on P4:PC61BM (1:1) blending with thermal treatment at 140 °C showed a V_oc_ of 0.86 V, a J_sc_ of 5.02 mA/cm^2^, and a FF of 0.29, giving a PCE of 1.24% which was sight lower than P3 [63]. These studies indicated that the monomer solubility and molecular weight of the polymers are crucial for electronic device application. IsoDPP containing polymers are good materials for BHJ solar cells if the molecular weight can be modified and the morphology can be improved. 

To obtain soluble polymers with high molecular weight, large scale alky chain substitution at the *N* position in isoDPP monomer core could be used. Upon Stille coupling and 4-methylundecane-alkylated isoDPP as monomer, a series of polymers (P8-13, Figure 5) with high molecular weight up to *M_n_* of 33.2 kDa and polymer dispersity index (PDI) of 2.77 were obtained [32]. All polymers exhibited LUMO energy levels around −3.5 eV, which was almost 0.3 eV higher than PC_71_BM, ensuring efficient exciton dissociation. These polymers showed broad UV/vis absorption in the visible with maxima from 533 to 679 nm. This matched well the sun light spectrum. P9 exhibited a μ_h_ as high as 0.03 cm^2^ V^−1^ s^−1^, whereas the mobilities of other polymers were lower, in the range of 10^−4^ cm^2^ V^−1^ s^−1^. The device performance of P9-based polymer was found V_oc_ of 0.76 V, J_sc_ of 10.28 mA/cm^2^, and FF of 0.65, giving a PCE of 5.1%, which indicated isoDPP was a promising acceptor building block for OPV. Studies regarding isoDPP based materials in solar cells were quite rare, among which, this was the best performance. Further studies on the devices using isoDPP polymers should be continued. 

For solar cell materials, the photostability is an important characteristic which notable influence the lifetime of device. H. Zhang et al. reported P14 and P15 (Figure 5) and studied the photostability of the isoDPP-based polymers. The structures of these two polymers were similar to P9 and P12 except that the *N*-alkylation of the isoDPP core was different [23]. After exposing the polymer solution under UV-light irradiation for one hour, the absorption spectrum was almost no change. This indicated the isoDPP-polymers showed good photostability, whereas polymers containing DPP unit in the main chain were easily to be photo-decomposed [67]. The photostability of the chromophore was significantly enhanced after exchanging the position between amide and carbonyl units. In this work, the authors also studied the electrochromism properties of the polymer. P14 showed a reversible electrochromism with isosbestic point near 710 nm, which indicated a red-shift of the absorption can be reached upon oxidation of the polymer backbone. Later, the same group using electrochemical polymerization methods obtained few isoDPP polymers, which also showed similar performance [68].

Except solar cells, isoDPP polymers are promising in OFETs. Very recently, X. Guo et al. reported copolymer containing isoDPP (P16, Figure 5) and DPP in the polymer backbone [69]. P16 afforded good order and quite close packing distance of 0.38 nm in the solid state, resulting it with good charge carrier transport ability. Bottom-gate, bottom-contact (BG,BC) OTFT was fabricated by drop-cast semiconductor onto the substrates, followed by annealing at 120 °C for 1 h, which exhibited an ambipolar transport like most DPP polymers. The OTFTs exhibited a high and balanced holes and electrons mobilities with values up to 0.02 cm^2^ V^−1^ s^−1^, which was 2–3 orders of magnitude higher than the reported DPP-based ‘homo’-polymer [70]. Later, H. Zhang et al. prepared P14 with weight molecular weight (*M_w_*) of 45.2 kDa. This polymer showed a μ_h_ of 0.09 [71]. Upon thiolation reaction by Lawesson regent, altering oxygen atom into sulfur atoms in the isoDPP core resulting isoDTPP, could not only alter the materials optical properties, but also change its electrochemical characteristics. IsoDTPP-based polymers showed red-shifted in the UV/vis absorption and narrow band gap compared to isoDPP-based polymers. In addition, the thiolated polymer showed enhanced ambipolar transporting ability with the μ_h_ increasing from 0.09 cm^2^ V^−1^ s^−1^ to 0.46 cm^2^ V^−1^ s^−1^, and an μ_e_ enchantment from non-detectable to 0.26 cm^2^ V^−1^ s^−1^. The thiolation reaction is a useful and simple method to optimize the frontier molecular orbitals and improve OFET performance. For isoDPP based polymers, optimizing the structure along the polymer backbone and developing new applications require significantly more research. 

### 3.2. Benzodipyrrolidone

**Figure 6:**
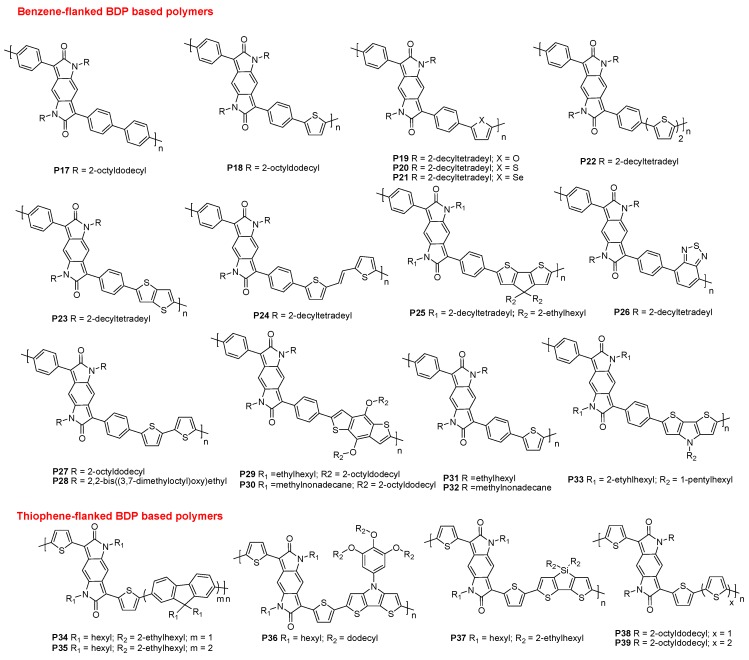
Chemical structure of polymers based on DPP.

Benzodipyrrolidone as a novel electron acceptor was successfully incorporated into several polymers. The performances of BDP based polymer electronic devices have been studied. Compared with isoDPP polymers, BDP polymers received more attention in the last few years. Through Suzuki coupling with good solubility in common organic solvents, F. Wudl et al synthesized the two BDP-containing polymers (P17 and P18, Figure 6) in 2011 [21]. The polymers showed good thermal stability of 5% weight loss occurring around 290 °C. P18 showed 90 nm red-shifted compared with isoDPP polymer with similar chemical structure [39]. This could be because BDP is a more strongly electron deficient than isoDPP, and shows a higher degree of backbone coplanarity. Compare to P17, in solution state, the maximum UV/vis absorption of P18 was 55 nm red-shifted (579 nm). This may be because thiophene is a stronger electron donating unit and favors intramolecular charge transfer. P17 BG,BC OFET exhibited n-channel performance with an μ_e_ of 2.4 × 10^−3^ cm^2^ V^−1^ s^−1^ after annealing at 240 °C, while the μ_e_ and μ_h_ of P18 was 6.4 × 10^−3^ and 3.5 × 10^−3^ cm^2^ V^−1^ s^−1^, respectively. The low charge transfer mobility of P18 could be ascribed to the low *M_n_* of only 8 kDa (PDI: 2.29). Later, Janssen et al. reported P18 with higher *M_n_* of 13.9 and PDI of 2.3 [72]. The P18 OTFT performance showed well-balanced μ_e_ and μ_h_ of 2.1 × 10^−1^ and 1.8 × 10^−1^ cm^2^ V^−1^ s^−1^, respectively, which showed the best performance for BDP based materials until now. The maximum of UV/vis absorption was 36 nm and 60 nm red-shifted in solution and thin film, respectively, compared with the one reported by Wudl [21]. The bathochromic shift was caused by that the high molecular weight improved intramolecular charge transfer mobility. KC Lee et al. reported the similar polymer P20 with even higher molecular weight with *M_n_* of 37 and PDI of 1.13 [73]. After annealing at 200 °C, this polymer showed an μ_e_ and a μ_h_ of only 1.1 × 10^−2^ and 1.2 × 10^−2^ cm^2^ V^−1^ s^−1^, respectively, which was significantly decreased compared with the P18 reported by Janssen [72]. The decreasing charge transport was possibly be related to the idea that the device was fabricated on glass substrate with a poly(methyl methacrylate) gate dielectric layer instead of Cytop dielectric and dielectric layer. To study the effects of actual ‘chalocogen atoms’ on OFET, the authors employed furan (Fu) and selenophene (Se) units into the polymer backbone, resulting polymers P19 and P21 (Figure 6). P20 and P21 had a rather crystalline domains with edge-on orientation, while P19 had a rather amorphous nature. The M. Du group reported that carriers migrate more easily along the path through crystalline grains than the amorphous film [74]. Among these three polymers, the highest charge transfer mobility was found in P21, which could be caused by the strongest electron-rich property of Se unit and more crystalline formation. 

McCulloch’s group reported a series of isoDPP-polymers (P22–26, Figure 6) [75]. The UV/vis absorption maxima of all polymers in thin film located between 620 and 650 nm except P25 and P26 being of 687 and 554 nm, respectively. The red shifting or blue shifting could be ascribed to the strong or weak push–pull and the intermolecular charge transfer band character of the polymers. In these polymers, P25 exhibited strongest push-pull effect. P23 exhibited the highest degree of crystallinity and a root mean square (RMS) roughness (1.82 nm) with the lowest μ_e_ of 1 × 10^−3^ cm^2^ V^−1^ s^−1^ among these polymers, while P25 showed the lowest RMS roughness at 0.4 nm, but highest μ_e_ of 1 × 10^−2^ cm^2^ V^−1^ s^−1^. This indicated that the polymer film surface roughness and push-pull effect could notable influence the charge carrier mobility. 

For the purpose to reduce the LUMO energy levels of polymers based on BDP, Zhang et al. reported an effectively strategy, which introduce the *N*-acylated BDP instead of *N*-alkylated BDP into the backbone of polymers (P29–P32, Figure 6) [37]. The studies showed that the *N*-acylated polymers (P30, P32) exhibit a LUMO level reduction of 0.3 eV and a 40 nm red-shift in the absorption compared with *N*-alkylated polymers (P29, P31). The *N*-acylated polymer P32 exhibited a good OFET device performance with an μ_e_ of 1.2 × 10^−3^ cm^2^ V^−1^ s^−1^ while the *N*-alkylated version (P31) showed 5.34 × 10^−4^ cm^2^ V^−1^ s^−1^. This novel method (*N*-acylation instead of *N*-alkylation) is not limited to BDP building block and can be applied to other chromophores containing five-membered lactam unit such as DPP, NDP and isodindigo. Later, alky- and acetal-type side chain-substituted BDP polymers were compared and studied (P27, P28, Figure 6). The alkylated polymer (P27) showed a μ_h_ of 1.8 × 10^−2^ cm^2^ V^−1^ s^−1^ after annealing at 120 °C for 5 min, while the polar acetal side chain-substituted polymer (P28) exhibited an optimal μ_h_ of 2.6 × 10^−2^ cm^2^ V^−1^ s^−1^, which was almost 1.5 times larger than P27 [76]. The solubility of P28 was improved compared to P27. This study indicated that an acetal-type side chain substitution could not only enhance the solubility of polymers, but also has significant impacts on the performance of OTFT. In a recent study, Zhang, et al. firstly reported the photostability of BDP-containing polymer. The polymer was quite stable under UV-light irradiation with *k* value of 0.68 h^−1^ [39]. The BDP-based polymers seem less stable than the isoDPP-based polymers [26], but are more stable than the DPP ones [67].

High charge mobility, low HOMO energy levels, and small band gaps, combined the broad UV/vis absorption render the BDP-based polymers interesting as building blocks for organic solar cells. However, there have only two articles reported the solar cells performance based on BDP-based polymer (P33, Figure 6), which showed PCE of 2.6% with a J_sc_ of −7.87 mA cm^−2^, a FF 44.6% and a V_oc_ of 0.74 V (OPV structure: ITO/PEDOT:PSS/P33:PCBM(1:2)/-LiF/Al) [77]. However, M. Yu et al. predicted the PCE of substituted phenyl-flanked BDP-based polymers could reach up to 10% through computation, which shows that the BDP-based polymers are great candidates for application in high performance OSCs [78]. Further studies on solar cells device based BDP polymers, typically the thienyl-flanked ones, should be continued soon. Compared to the polymer containing isoDPP units, the BDP versions show lower LUMO and HOMO levels, smaller band gap, and bathochromic shift in the UV/vis absorption.

In order to obtain a broad absorption in the near-infrared region even infrared region of low band gap polymers, thiophene-flanked BDP was introduced to the backbone of polymers forming P34 to P39 [43,44]. The polymers exhibited UV/vis absorption maxima between 640 and 1006 nm in the solid state, an optical band gaps between 0.79 and 1.27 eV and 5% weight loss occurring around or above 400 °C. P38 OFETs showed a good μ_h_ and μ_e_ of 0.2 and 0.1 cm^2^ V^−1^ s^−1^, respectively, while the value of P39 was 0.08 and 0.01 cm^2^ V^−1^ s^−1^ respectively. Such high charge mobility combined a strong overlap with the solar radiation spectrum renders the thiophene-flanked BDP polymers suitable for solar cells application. 

### 3.3. Naphthodipyrrolidone

**Figure 7:**
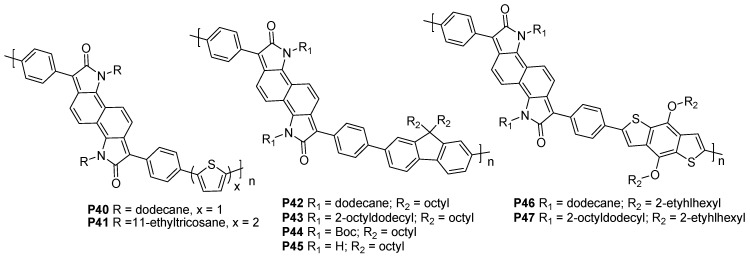
Chemical structure of polymers based on NDP.

Polymers containing NDP unit in the main chain have received the least attention among the three reviewed chromophore-polymers, which is likely due to the fact that the NDP pigment was not developed until very recently (2014). Until now, only four articles have reported the synthesis and properties of NDP-based polymers. The first NDP-polymer P40 (Figure 7) was prepared upon Stille coupling of symmetric di-*N*-alkylated 3,8-di(4-bromophenyl)-NDP (NDP-2) and 2,5-bis-(trimethylstannyl)thiophene with *M_n_* of 7.5 kDa and PDI of 2.2 [20]. P40 is soluble in common organic solvents showing a blue color. The maximum absorption of P40 was located at 615 in solution with extinction coefficient of 2.8 × 10^4^ L mol^−1^ cm^−1^, which was red-shifted almost 126 and 36 nm compared by the isoDPP- and BDP-based polymers (P17) with similar in chemical structure, respectively [19]. The bathochromic shift could be ascribed the NDP core exhibits the largest π-conjugation in these three chromophores.

Later, the same group reported another four D–A copolymers constructed by NDP as acceptor, and fluorine (P42, P43) or benzo[1,2-b:4,5-]dithiophene (BDT, P46, P47) as donor units [46]. All the polymers exhibited broad optical absorption between 400 nm and 1000 nm, and a narrow band gap (1.30 to 1.60 eV). Compared to the fluorine-based polymers, the BDT-based materials showed bathochromic shift in the UV/vis absorption and smaller electrochemical band gap. This could be ascribed to the stronger D–A interaction formed in the BDT polymers, which resulted in improved intramolecular charge transfer within the polymer backbone. The photostability of the NDP-base polymers was studied in this work. During the light irradiation, polymers solution was extremely stable under ambient light condition but easily decomposed during the UV light irradiation. In the solid state, the polymers were stable even exposing UV light. Recently, Y. Zhu’s group synthesized Boc-substituted NDP polymer (P44, Figure 7) and used it to construct OFET, which showed n-type behavior with an μ_e_ of 2.4 × 10^−4^ cm^2^ V^−1^ s^−1^ [79]. After thermal annealing, the Boc unit could be decomposed, while hydrogen-bonded (NH...OC) crosslinked polymers were formed (P45). The hydrogen bonded polymer showed an μ_e_ of up to 0.01 cm^2^ V^−1^ s^−1^, which was almost 40 times higher than the P44. The improvement charge transfer value could be ascribed to the hydrogen-bonding network formed between the amide and carbonyl unit, which not only shortened the distance between the neighboring polymer backbones, but also favored the formation of an ordered structure [80,81,82]. Soon after, the same group used P47 to construct OFET showing ambipolar semiconducting behavior with a μ_h_ of 0.015 cm^2^ V^−1^ s^−1^ and an μ_e_ of 0.227 cm^2^ V^−1^ s^−1^. In this work, another polymer (P41) using bithiophene as donor unit was synthesized, which showed an μ_e_ of even up to 0.667 cm^2^ V^−1^ s^−1^ [83]. The charge carrier mobility was only one order magnitude lower when the device was exposed in air after one week. In most cases, the n-type organic FETs are easy to be doped and sensitive in ambient environment, implying good stability. The NDP chromophore is a promising acceptor building block in building high performance n-type semiconductors. Due to the NDP-based polymers shows broad optical absorption, narrow band gap and high charge carrier mobility, they show good potential for solar cells. The optical and electrochemical properties, as well as electronic device performance based on the part of the three small molecules are summarized in Table 2. 

## 4. Conclusions and Outlook

In this article, small molecules and polymers based on high-performance pigments such as isoDPP, BDP, and NDP have been reviewed. These pigments are similar in chemical structure and show similar properties. The three reviewed chromophore exhibit electron-deficient characteristic. Using these monomers for polymerization, the obtained polymers exhibit often deep color with high extinction coefficient and extremely weak luminescent with quantum yields below the detection limit. The cores of the compounds are coplanar, which ensure full conjugation. After *N*-substitution, these pigments with bi-bromine atoms exhibit good solubility properties in common organic solvents, and are thus suitable for polymerization. For the *N*-substitution, *N*-acylation instead of *N*-alkylation can reduce the LUMO levels of the compounds (P 29 and 30; P31 and 32). Acetal-type side chains can also impact polymer OFET performance (P27 and P28). The substituted Boc unit on the amide group can be decomposed upon thermal annealing while hydrogen bonds are forming. The formed hydrogen bonds reassemble the molecular packing, leading to more ordered structures and cross-linking-liked materials are formed, which result in notable improvements in electronic device performance (BDP 6 and 7; P44 and 45). However, the polymer solubility can be affected when Boc-substituted pigments are used for polymerization.

Regarding the synthesis of the monomers, thiophene-flanked isoDPP and BDP have been developed. The UV/vis absorption maxima of thienyl-flanked isoDPP or BDP chromophores exhibit red shifting of almost 50 nm (between isoDPP 4 and isoDPP 5) or 101 nm (between BDP 2 and BDP 13) compared to the phenyl-flanked versions, respectively. This can be ascribed to the fact that the thienyl unit is a more electron-rich unit compared with the phenyl unit, and a D–A system formed, which favors a bathochromic shift. In thienyl-substituted chromophores, due to the sulfur-oxygen interactions, the thienyl-flanked pigments show planar structures. Extending coplanar structures will lead to strong π–π stacking, bathochromic shifts, and lower LUMO values (BDP 8 to 11). Polymers using the thiophene-flanked chromophore as the monomer often show broad optical absorption, a strong push-pull system, and good planarity in the backbone, where each property favors good performance in electronic devices. It is desirable to further optimize the chemical structure of these pigments, such as by using different electron-rich capability units, including furan, selenophene, and thieno[3,2-b]thiophene, instead of phenyl or thiophene units to flank the core of these chromophores. 

π-Conjugation extension can alter the material properties and improve pigments electronic device performance. Even though the structures among isoDPP, BDP, and NDP are similar, their optical properties, as well as their molecular packing and stability, are different with respect to core extension. For instance, (i) The UV/vis maxima exhibit a bathochromic shift in order from isoDPP (348 nm, isoDPP 4) to BDP (458 nm, BDP 2), and NDP (567 nm, NDP 2). This can be ascribed to a larger π-conjugated extension; (ii) The core extension leads to molecules with short distances between the adjacent molecules (3.5 Å for isoDPP 4, 3.56 Å for BDP 2, and 3.38 Å for NDP 2), which favors strong π–π stacking and increased charge transfer mobility; (iii) The chromophore stability decreased during the core-extension. IsoDPP shows good stability under UV light, while the NDP is easily decomposed in the solution state; (iv) With π-conjugation extension, the charge mobility can be significantly improved and the OFET channel can be altered. NDP-based materials often exhibit n-type or ambipolar characteristics, while isoDPP and BDP show p-channel characteristics. 

For this field of research, further enlargement of the core of the chromophore or flanked units has become favored. Compared with DPP-based materials, materials based on the reviewed chromophore are significantly less studied. With further structure optimization, more developments in applications, such as organic solar cells, OFETs, and dopant-free hole transfer in perovskite solar cells, can become both desirable and attainable. In conclusion, this article reviewed polymers based on isoDPP, BDP, and NDP chromophores, where generalized insights into structure−property relationships of different heteroaromatic blocks in bislactam-based D−A polymers were given, which should facilitate the continued design of high-performance polymers for the promotion in π-conjugated materials applications.

## Figures and Tables

**Table 1:** List of properties of small molecules based on isoDPP, BDP, and NDP.

Polymers	π-π Stacking Distance Å	UV/vis [nm] In Solution/In Thin Film	Band Gap [eV] E_g_^opt^/E_g_^ec^	μ_e_ cm^2^ V^−1^ s^−1^	μ_h_ cm^2^ V^−1^ s^−1^	PCE [%]	Ref.
**IsoDPP1**	--	352,534/356,512	1.93/1.68	--	--	1.26	[28]
**IsoDPP2**	--	352,533/352,519	1.90/1.66			1.32	[28]
**IsoDPP3**	--	352,534/356,516	1.90/1.68	--	--	1.56	[28]
**IsoDPP4**	3.5	--	--	--	--	--	[29]
**IsoDPP5**	3.7	--	--	--	--	--	[29]
**IsoDPP7**	6.969	437/--	--	--	--	--	[30]
**IsoDPP8**	--	--	2.78/2.65	--	--	--	[32]
**IsoDPP9**	3.34	--	--	--	--	--	[32]
**BDP1**	3.56	458/--	--/2.20	--	--	--	[21]
**BDP2**	--	458/523	--/2.37	--	--	--	[21]
**BDP3**	--	478/531	2.05/--	--	--	--	[37]
**BDP4**	--	478/504,555	2.00/--	--	--	--	[37]
**BDP6**	--	463,519/--		--	8.21 × 10^−2^		[40]
**BDP7**	--	463/--		--	1.8 × 10^−3^	--	[40]
**BDP9**	3.3	583,630/--	1.79/1.30	--	--	--	[45]
**BDP13**	3.29	559/593	1.38/1.67	--	--	--	[43]
**NDP2**	3.38	567/--	1.46/1.48	1.0 × 10^−2^	--	--	[74]

E_g_^opt^: optical band gap; E_g_^ec^: electrochemical band gap; --: not available.

**Table 2:** List of properties of polymers based on isoDPP, BDP, and NDP.

Polymers	*M_n_* [kDa]/PDI	UV/vis [nm] In Solution/In Thin Film	Band Gap [eV] E_g_^opt^/E_g_^ec^	μ_e_ cm^2^ V^−1^ s^−1^	μ_h_ cm^2^ V^−1^ s^−1^	PCE [%]	Ref.
**P1**	7.6/1.3	328, 409/	1.94/1.95	--	--	--	[19]
**P2**	3.4/1.4	331, 360/	2.07/1.83	--	--	--	[19]
**P3**	10.1/1.9	545/548	1.83/1.87	2.0 × 10^−5^	--	2	[64]
**P4**	2.3/2.0	585/--	--	--	--	1.24	[66]
**P5**	1.7/1.9	584/480	1.71/	--	--	0.23	[66]
**P9**	21.1/1.5	569/642	1.59/1.99	--	3.4 × 10^−2^	5.1	[32]
**P16**	73.0/5.1	751,785/756,825	1.23/1.66	2.0 × 10^−2^	2.0 × 10^−2^	--	[69]
**P17**	19.1/2.1	524/538	1.9/--	2.4 × 10^−3^	--	--	[21]
**P18**	7.1/2.3	579/579	1.68/--	6.4 × 10^−3^	3.5 × 10^−3^	--	[21]
	13.9/2.3	615/639	1.69/1.72	1.8 × 10^−1^	2.1 × 10^−1^	--	[72]
**P19**	25.0/1.6	602/622	1.66/--	7.0 × 10^−3^	--	--	[73]
**P20**	37.0/1.1	611/630	1.67/1.76	1.1 × 10^−2^	1.2 × 10^−2^	--	[73]
**P21**	74.0/1.7	611/652	1.64/1.73	1.9 × 10^−2^	1.7 × 10^−2^	--	[73]
**P22**	17.0/2.5	602/624	1.58/--	1.0 × 10^−3^	--	--	[75]
**P23**	15.0/3.4	613/631	1.61/--	1.0 × 10^−3^	--	--	[75]
**P24**	20.0/2.3	622/647	1.49/--	2.0 × 10^−3^	--	--	[75]
**P25**	21.0/2.0	661/687	1.49/--	1.0 × 10^−2^	--	--	[75]
**P26**	24.0/1.3	543/554	1.85/	1.0 × 10^−2^	--	--	[75]
**P31**	49.0/2.5	621/632	1.56/2.04	5.3 × 10^−4^	--	--	[37]
**P32**	23.0/2.3	645/673	1.44/1.72	1.2 × 10^−2^	--	--	[37]
**P38**	34.0/1.7	--/960	1.03/--	1.0 × 10^−1^	2.0 × 10^−1^	--	[44]
**P40**	7.6/2.2	615/678	1.38/1.41	--	--	--	[20]
**P41**	14.9/2.8	675/703	1.39/1.47	6.7 × 10^−1^	7 × 10^−3^		[83]
**P44**	18.7/2.1	626/640	1.57/1.68	2.0 × 10^−4^	--	--	[79]
**P45**	--	--/665	1.46/1.48	1.0 × 10^−2^	--	--	[79]

E_g_^opt^: optical band gap; E_g_^ec^: electrochemical band gap; --: not available.

## Appendix A

**Zhifeng Deng** received his M.S. degree from Qingdao University of Science and Technology in 2010. After that, he worked in Yokohama tire co., LTD. as material engineer for 3 years, and had been to Hirsuka Research and Development Center (Japan) for further study in 2012. He is now a PhD candidate of Northwestern Polytechnical University, and working as a lecturer at the School of Material Science and Engineering, Shaanxi University of Technology. His work is on synthesis and characterization of novel DPP-based derivatives for organic field effect transistors.

**Prof. Taotao Ai** received his PhD degree in Materials Physics and Chemistry from Shaanxi University of Science and Technology, China, in 2015. He had been to Johns Hopkins university, New Jersey institute of technology, Rutgers University and Pennsylvania foundry association for academic visiting in 2016. He is currently a professor at the School of Material Science and Engineering, Shaanxi University of Technology. His research interests include flexible optoelectronic materials and devices. Prof. Ai has published more than 150 papers in international journals and have been awarded 20 Chinese patents.

**Prof. Huiling Du** received her Ph.D degree in Electronic Science and Technology from Xi’an Jiaotong University in 2002. After that, she worked in UC-Davis (USA) as a visiting scholar academic visitor for one year. She is currently a professor at the School of Materials Science and Engineering, Xi’an University of Science and Technology. Her research interests include development novel functional materials for energy conversion and storage, electronic device, gas adsorption/separation, clean and renewable energy and so on. Prof. Du has published around 150 papers in international journals and been awarded 18 Chinese patents. Prof. Du is now co-editor of *Material Reports*, *Journal of Functional Materials* and *Journal of Xi’an University of Science and Technology*.

**Prof. Haichang Zhang** received his Ph.D degree in Department of Physical chemistry, University of Cologne (Germany) in 2014 supervised by Prof. Bernd Tieke. After that, he worked as a postdoctoral research assistant in University of Bordeaux (France), University of Akron (USA) and University of Cologne, separately. He is currently a professor at Qingdao University of Science and Technology (China). His research interests include new D-A type π-conjugated materials and their applications in electronic device and coatings. Prof. Zhang has published 44 papers in international journals.

## References

[1] Eom S.H., Nam S.Y., Do H.J., Lee J., Jeon S., Shin T.J., Jung I.H., Yoon S.C., Lee C. (2017). Dark current reduction strategies using edge-on aligned donor polymers for highly detectivity and responsivity organic photodetectors. Polym. Chem..

[2] Song H., Deng Y., Jiang Y., Tian H., Geng Y. (2018). π-Conjugation expended isoindigo derivatives and the donor–acceptor conjugated polymers: Synthesis and characterization. Chem. Commun..

[3] Coakley K.M., Mcgehee M.D. (2004). Conjugated polymer photovoltaic cells. Chem. Mater..

[4] Guo X., Facchetti A., Marks T.J. (2014). Imide- and amide-functionalized polymer semiconductors. Chem. Rev..

[5] Wu T., Yu C., Guo Y., Liu H., Yu G., Fang Y., Liu Y. (2012). Synthesis, structure, and properties of thieno[3,2-*b*]thiophene and dithiophene bridged isoindigo derivatives and their organic field-effect transistors performance. J. Phys. Chem. C.

[6] Chen S., Sun B., Guo C., Hong W., Meng Y., Li Y. (2014). 3,3′-(Ethane-1,2-diylidene)bis(indolin-2-one) based conjugated polymers for organic thin film transistors. Chem. Commun..

[7] Kim I., Jung J.-E., Lee W., Park S., Kim H., Jho Y.-D., Woo H.Y., Kyhm K. (2019). Two-step energy transfer dynamics in conjugated polymer and dye-labeled aptamer-based potassium ion detection assay. Polymers.

[8] Gao C., Wang L., Li X., Wang H. (2014). Rational design on D–A conjugated P(BDT-DTBT) polymers for polymer solar cells. Polym. Chem..

[9] Ni W., Wan X., Li M., Wang Y., Chen Y. (2015). A-D—A small molecules for solution-processed organic photovoltaic cells. Chem. Commun..

[10] Shiau S., Chang C.-H., Chen W.-J., Wang H.-J., Jeng R.-J., Lee R.-H. (2015). Star-shaped organic semiconductors with planar triazine core and diketopyrrolopyrrole branches for solution-processed small-molecule organic solar cells. Dye Pigments.

[11] Chen W.-J., Chen Y.-C., Kuo D.-W., Chen C.-T., Liu B.-T., Jeng R.-J., Lee R.-H. (2018). A star-shaped conjugated molecule featuring a triazole core and diketopyrrolopyrrole branches is an efficient electron-selective interlayer for inverted polymer solar cells. RSC Adv..

[12] Lee R.-H., Yang L.-C., Wu J.-Y., Jeng R.-J. (2017). Synthesis of di(ethylene glycol)-functionalized diketopyrrolopyrrole derivative-based side chain-conjugated polymers for bulk heterojunction solar cells. RSC Adv..

[13] Oh J.Y., Rondeau-Gagne S., Chiu Y.-C., Chortos A., Lissel F., Wang G.-J.N., Schroeder B.C., Kurosawa T., Lopez J., Katsumata T. (2016). Intrinsically stretchable and healable semiconducting polymer for organic transistors. Nature.

[14] Yao J., Yu C., Liu Z., Luo H., Yang Y., Zhang G., Zhang D. (2016). Significant improvement of semiconducting performance of the diketopyrrolopyrrole-quaterthiophene conjugated polymer through side-chain engineering via hydrogen-bonding. J. Am. Chem. Soc..

[15] Jung J.W., Jo W.H. (2015). Alow band-gap copolymer composed of thienyl substituted anthracene and diketopyrrolopyrrole compatible with multiple electron acceptors for high efficiency polymer solar cells. Polym. Chem..

[16] Sugiyama F., Kleinschmidt A.T., Kayser L.V., Rodriquez D., Finn M., Alkhadra M.A., Wan J.M.-H., Ramiez J., Chiang A.S.-C., Root S.E. (2018). Effects of flexibility and branching of side chain on the mechanical properties of low-bandgap conjugated polymers. Polym. Chem..

[17] Falzon M.-F., Zoombelt A.P., Winek M.M., Janssen R.A.J. (2011). Diketopyrrolopyrrole-based acceptor polymers for photovoltaic application. Phys. Chem. Chem. Phys..

[18] Wuckelt J., Doering M., Langer P., Goerls H., Beckert R. (1997). Studies of mild dehydrogenations in heterocyclic systems. Tetrahedron Lett..

[19] Welterlich I., Charov O., Tieke B. (2012). Deeply colored polymers containing 1,3,4,6-tetraarylpyrrolo[3,2-\r b\r]pyrrole-2,5-dione (isoDPP) units in the main chain. Macromolecules.

[20] Zhang H., Neudörfl J.-M., Tieke B. (2014). 1,6-Naphthodione-based monomers and polymers. Polym. Chem..

[21] Cui W., Yuen J., Wudl F. (2011). Benzodipyrrolidones and their polymers. Macromolecules.

[22] Crossley D.L., Goh R., Cid J., Vitorica-Yrezabal I., Turner M., Ingleson M.J. (2017). Borylated arylamine-benzothiadiazole donor-acceptor materials as low-LUMO, low-band-gap chromophores. Organometallics.

[23] Rochat A.C., Iqbal A., Pfenninger J., Casser L. (1985). Process for Dyeing a High Molecular Organic Material, Polycyclic Compounds and Their Preparation. EP 016 3609.

[24] Fuerstenwerthm H. (1987). Heerocyclische Vrindungen. DE 3 525 109 A1.

[25] Langer P., Wuckelt J., Doering M. (2000). New and efficient synthesis of pyrrolo[3,2-b]pyrrole-2,5-diones by double-anion-capture reactions of ester carbanions with Bis(imidoyl)chlorides of oxalic acid. J. Org. Chem..

[26] Kirkus M., Knippenberg S., Beljonne D., Cornil J., Jansen R.A.J., Meskers S.C. (2013). Synthesis and optical properties of pyrrolo[3,2‑*b*]pyrrole2,5(1*H*,4*H*)‑dione (iDPP)-based molecules. J. Phys. Chem. A.

[27] Guo E.Q., Ren P.H., Zhang Y.L., Zhang H.C., Yang W.J. (2009). Diphenylamine end-capped 1,4-diketo-3,6-diphenylpyrrolo[3,4-*c*]pyrrole (DPP) derivatives with large two-photon absorption cross-sections and strong two-photon excitation red fluorescence. Chem. Commun..

[28] Song S., Ko S.-J., Shin H., Jin Y., Kim I., Kim J.Y., Suh H. (2013). Pyrrolo[3,2-*b*]pyrrole based small molecules as donor materials for OPVs. Sol. Energy Mater. Solar Cells.

[29] Gendron D., Gann E., Pattison K., Maasoumi F., Mcneill C.R., Watkins S.E., Burn P.L., Powell B.J., Shaw P.E. (2014). Synthesis and properties of pyrrolo[3,2-*b*]pyrrole-1,4-diones (isoDPP) derivatives. J. Mater. Chem. C.

[30] Zhang H., Welterlich I., Neudoerfl J.-M., Tieke B., Yang C., Chen X., Yang W. (2013). Synthesis and characterization of 1,3,4,6-tetraarylpyrrolo[3,2-*b*]-pyrrole-2,5-dione (isoDPP)-based donor–acceptor polymers with low band gap. Polym. Chem..

[31] Endo A., Matsumoto S., Mizuguchi J. (1999). Correlation between electronic and crystal structure in diketopyrrolopyrrole pigments. Proc. Nip Digit. Fabr. Conf..

[32] Lu S., Drees M., Yao Y., Boudinet D., Yan H., Pan H., Wang J., Li Y., Usta H., Facchetti A. (2013). 3,6-Dithiophen-2-yl-diketopyrrolo[3,2-*b*]pyrrole (isoDPPT) as an acceptor building block for organic opto-Electronics. Macromolecules.

[33] Naik M.A., Venkatramaiah N., Kanimozhi C., Patil S. (2012). Influence of side-chain on structural order and photophysical properties in thiophene based diketopyrrolopyrroles: A systematic study. J. Phys. Chem. C.

[34] Greenhalgh C.W., Carey J.L., Newton D.F. (1980). The synthesis of quinodimethanes in the benzodifuranone and benzodipyrrolidone series. Dyes Pigments.

[35] Greenhalgh C.W., Carey J.L., Hall N., Newton D.F. (1994). The benzodifuranone chromogen and its application to disperse dyes. J. Soc. Dyes Colour..

[36] Kuwabara J., Yamagata T., Kanbara T. (2010). Solid-state structure and optical properties of highly fluorescent diketopyrrolopyrrole derivatives synthesized by cross-coupling reaction. Tetrahedron.

[37] Deng P., Liu L., Ren S., Li H., Zhang Q. (2012). *N*-acylation: An effective method for reducing the LUMO energy levels of conjugated polymers containing five-membered lactam units. Chem. Commun..

[38] Zhang H., Ghasimi S., Tieke B., Schade A., Spange S. (2014). Aminobenzodione-based polymers with low bandgaps and solvatochromic behavior. Polym. Chem..

[39] Ling Y., Xiang C., Zhou G. (2017). Multicolored electrochrmosim from benzodipyrrolidone-based ambipolar electrochromes at a fixed potential. J. Mater. Chem. C.

[40] Zhang H., Deng R., Wang J., Li X., Chen Y.-M., Liu K., Taubert C., Cheng S.Z.D., Zhu Y. (2017). Crystalline organic pigment-based field-effect transistors. ACS Appl. Mater. Interfaces.

[41] Li Y., Li C., Zhang L., Zhou W. (2019). Effect of microscopic-ordered structures on intrinsic thermal conductivity of liquid-crystalline polysiloxane. J. Mater. Sci. Mater. Electron..

[42] Li Y., Zhang L., Chang M., Chang M. (2014). Cholesteric cyclohexane-containing side-chain liquid-crystalline polysiloxanes. Mater. Sci. Forum.

[43] Cui W., Wudl F. (2013). Dithienylbenzodipyrrolidone: New Acceptor for Donor–Acceptor Low Band Gap Polymers. Macromolecules.

[44] Rumer J.W., Levick M., Dai S.-Y., Rossbauer S., Huang Z., Binik L., Anthopoulos T.D., Durrant J.R., Procter D.J., McCulloch I. (2013). BPTs: Thiophene-flanked benzodipyrrolidone conjugated polymers for ambipolar organic transistors. Chem. Commun..

[45] Wang Y., Chen L., El-Shishawy R.M., Aziz S.G., Mulen K. (2014). Synthesis and optophysical properis of dimeric aza-bodipy dyes with a push-pull benzodipyrrolidone core. Chem. Commun..

[46] Zhang H., Ying S., Tieke B., Zhang J., Yang W. (2015). 1,6-Naphthodipyrrolidone-based donor–acceptor polymers with low band gap. Polymer.

[47] Deng Z., Hao X., Zhang P., Li L., Yuan X., Ai T., Bao W., Kou K. (2019). Donor-acceptor molecules based on diketopyrrolopyrrole, benzodipyrrolidone and naphthodipyrrolidone: Organic crystal field-effect transistors. Dye Pigments.

[48] Guenes S., Neugebauer H., Sariciftci N.S. (2007). Conjugated Polymer-Based Organic Solar Cells. Chem. Rev..

[49] Chen J., Hsu C.-S. (2011). Conjugated polymer nanostructures for organic solar cell applications. Polym. Chem..

[50] Kini G.P., Choi J.Y., Jeon S.J., Suh S., Moon D.K. (2019). Effects of incorporated pyrazine on the interchain packing and photovoltaic properties of wide-bandgap D–A polymers for non-fullerene polymer solar cells. Polym. Chem..

[51] Hou W., Xiao Y., Han G., Lin J.-Y. (2019). The applications of polymers in solar cells: A review. Polymers.

[52] Tong J., An L., Lv J., Guo P., Wang X., Yang C., Xia Y. (2019). Enhanced photovoltaic performance in D-π-A copolymers containing triisopropylsilylethynyl-substituted dithienobenzodithiophene by modulating the electron-deficient units. Polymers.

[53] Zhang D., Hu R., Cheng J., Chang Y., Huo M., Yu J., Li L., Zhang J.-P. (2018). Appropriate donor–acceptor phase separation structure for enhancement of charge generation and transport in polymer solar cells. Polymers.

[54] Guo X., Hao X., Kim F.S., Liyanage A.D.T., Jenekhe S.A., Watson M.D. (2011). Thieno[3,4-*c*]pyrrole-4,6-dione-based donor–acceptor conjugated polymers for solar cells. Macromolecules.

[55] Zhang H., Liu M., Yang W., Judin L., Hukka T.I., Priimagi A., Deng Z., Vivo P. (2019). Thionation enhances the performance of polymeric dopant-free hole-transfer materials for perovskite solar cells. Adv. Mater. Interfaces.

[56] Zhu X., Lu K., Xia B., Fang J., Zhao Y., Zhao T., Wei Z., Jiang L. (2016). Improving the performance of random copolymer based organic solar cells by adjusting the film features of active layers using mixed solvents. Polymers.

[57] Williams E.L., Ang T.S., Ooi Z., Sonar P., Lin T.T., Neo W.T., Song J., Hobley J. (2015). Optical characterization of the hole polaron in a series of diketopyrrolopyrrole polymers used for organic photovoltaics. Polymers.

[58] Ponnappa S.P., Liu Q., Umer M., MacLeod J., Jickson J., Ayoko G., Shiddiky M.J.A., O’Mullane A.P., Sonar P. (2019). Naphthalene flanked diketopyrrolopyrrole: A new conjugated building block with hexyl or octyl alkyl side chain for electropolymerization studies and its biosensor applications. Polym. Chem..

[59] Lee S.M., Lee H.R., Dutta G.K., Lee J.H., Yang C. (2019). Furan-flanked diketopyrrolopyrrole-based chalcogenophene copolymers with siloxane hybrid side chain for organic field-effect transistors. Polym. Chem..

[60] Li Y., Sonar P., Murphy L., Hong W. (2013). High mobility diketopyrrolopyrrole (DPP)-based organic semiconductor materials for organic thin film transistors and photovoltaics. Energy Environ. Sci..

[61] Zhang K., Tieke B. (2008). Highly luminescent polymers containing the 2,3,5,6-tetraarylated pyrrolo[3,4-*c*]pyrrole-1,4-dione (*N*-Aryl DPP) chromophore in the main chain. Macromolecules.

[62] Wang Z.-S., Koumura N., Cui Y., Takahashi M., Sekiguchi H., Mori A., Kubo T., Furube A., Hara K. (2008). Hexylthiophene-functionalized carbazole dyes for efficient molecular photovoltaics: Tuning of solar-cell performance by structure modification. Chem. Mater..

[63] Liu X., Wen W., Bazan G.C. (2012). Post-deposition treatment of an arylated-carbazole conjugated polymer for solar cell fabrication. Adv. Mater..

[64] Song S., Ko S.-J., Shin H., Jin Y., Kim I., Kim J.Y., Suh H. (2012). Synthesis of the pyrrolo[3,2-*b*]pyrrole-based copolymer with enhanced open circuit voltage. Synth. Met..

[65] Zhu Y., Rabindranath A.R., Beyerlein T., Tieke B. (2007). Highly luminescent 1,4-diketo-3,6-diphenylpyrrolo[3,4-*c*]pyrrole-(DPP-) based conjugated polymers prepared upon suzuki coupling. Macromolecules.

[66] Gironda R., Choi J.W., Miozzo L., Tondelier D., Papagni A., Yassar A. (2014). A novel isomer of pyrrolo[3,4-*c*]pyrrole-1,4(2*H*, 5*H*)-dione as a new building block for polymer solar cells. Synth. Met..

[67] Beyerlein T., Tieke B. (2000). New photoluminescent conjugated polymers with 1,4-dioxo-3,6-diphenylpyrrolo[3,4-*c*]pyrrole (DPP) and 1,4-phenylene units in the main chain. Macromol. Rapid Commun..

[68] Welterlich I., Neudorfl J.M., Tieke B. (2015). Electrochemical polymerization of 1,3,4,6-tetraarylpyrrolo[3,2-*b*]pyrrole-2,5-dione (isoDPP) derivatives. Polym. Chem..

[69] Guo X., Puniredd S.R., He B., Marszalek T., Baumgarten M., Pisula W., Mullen K. (2014). Combination of two diketopyrrolopyrrole isomers in one polymer for ambipolar transport. Chem. Mater..

[70] Zoombelt A.P., Mathijssen S.G.J., Turbiez M.G.R., Wienk M.M., Janssen R.A.J. (2010). Small band gap polymers based on diketopyrrolopyrrole. J. Mater. Chem..

[71] Zhang H., Yang K., Zhang K., Zhang Z., Sun Q., Yang W. (2018). Thionating iso-diketopyrrolopyrrole-based polymers: From p-type to ambipolar field effect transistors with ehanced charge mobility. Polym. Chem..

[72] Pruissen G.W.P., Pidko E.A., Wienk M.M., Jassen R.A.J. (2014). High balanced ambipolar charge carrier mobility in benzodipyrrolidone conjugated polymers. J. Mater. Chem. C.

[73] Lee K.C., Park W.-T., Noh Y.-Y., Yang C. (2014). Benzodipyrrolidone (BDP) based polymer semiconductors containing a series of chalcogen atoms: Comparehensive investigation of effect of heteroaromatic blocks on intrinsic semiconducting properties. ACS Appl. Mater. Interfaces.

[74] Du H., Ma C., Ma W., Wang H. (2018). Microstructure evolution and dielectric properties of Ce-doped SrBi_4_TiO_15_ ceramics synthesized via glycine-nitrate process. Process. Appl. Ceram..

[75] Rumer J.W., Rossbauer S., Planells M., Watkins S.E., Anthopoulos T.D., McCulloch I. (2014). Reduced roughness for improved mobility in benzodipyrrolidone-based, n-type OFETs. J. Mater. Chem. C.

[76] Hong W., Guo C., Li Y., Zheng Y., Huang C., Lu S., Facchetti A. (2012). Synthesis and thin-film transistor performance of benzodipyrrolinone and bithiophene donor–acceptor copolymers. J. Mater. Chem..

[77] Yue W., Huang X., Yuan J., Ma W., Krebs F.C., Yu D. (2013). A novel benzodipyrrolidone-based low band gap polymer for organic solar cells. J. Mater. Chem. A.

[78] Yu M., Zhang L., Peng Q., Zhao H., Gao J. (2015). Narrow-band gap benzodipyrrolidone donor conjugated polymer: A theoretical investigation. Comput. Theor. Chem..

[79] Zhang H., Yang K., Chen Y.-M., Bhatta R., Tsige M., Cheng S.Z.D., Zhu Y. (2017). Polymers based on benzodipyrrolidone and naphthodipyrrolidone with latent hydrogen-bonding on the main chain. Macroml. Chem. Phys..

[80] Zhang H., Liu K., Wu K.-Y., Chen Y.-M., Deng R., Li X., Jin H., Li S., Chuang S.S.C., Wang C.-L. (2018). Hydrogen-bonding-mediated solid-state self-assembled isoepindolidiones (isoEpi) crystal for organic field-effect transistor. J. Phys. Chem. C..

[81] Hu Y., Motzer H., Etxeberria A., Fernandez-Berridi M., Iruin J., Painter P.C., Coleman M.M. (2000). Concerning the self-association of *N*-vinyl pyrrolidone and its effect on the determination of equilibrium constants and the thermodynamics of mixing. Macromol. Chem. Phys..

[82] Kuo S.W., Chang F.C. (2001). Studies of miscibility behavior and hydrogen bonding in blends of Poly(vinylphenol) and poly-(vinylpyrrolidone). Macromolecules.

[83] Zhang H., Zhang S., Mao Y., Liu K., Chen Y.-M., Jiang Z., Strzalka J., Yang W., Wang C.-L., Zhu Y. (2017). Naphthodipyrrolidone (NDP) based conjugated polymers with high electron mobility and ambipolar transport properties. Polym. Chem..

